# Identifying key blood markers for bacteremia in elderly patients: insights into bacterial pathogens

**DOI:** 10.3389/fcimb.2024.1472765

**Published:** 2025-01-16

**Authors:** Shi-Yan Zhang, Ying Zhuo, Bu-Ren Li, Ying-Ying Jiang, Jie Zhang, Na Cai, Lin Yang

**Affiliations:** Department of Clinical Laboratory, Fuding Hospital, Fujian University of Traditional Chinese Medicine, Fuding, Fujian, China

**Keywords:** elderly, blood culture, neutrophil-to-lymphocyte ratio, ROC analysis, *Escherichia coli*, bacteremia

## Abstract

**Background:**

This study aimed to assess the distribution of bacteremia pathogens in elderly patients, examine the impact of gender on pathogen distribution, and evaluate the predictive value of routine blood parameters for diagnosing bacteremia.

**Methods:**

A retrospective analysis was conducted on 151 elderly patients (≥60 years old) admitted to Fuding Hospital, Fujian University of Traditional Chinese Medicine between October 2022 and June 2023. Comprehensive routine blood tests and blood cultures were performed. The diagnostic efficacy of routine blood parameters, including white blood cell (WBC), neutrophil-to-lymphocyte ratio (NLR), platelet-lymphocyte ratio (PLR), and red blood cell distribution width (RDW), was evaluated using receive operating characteristic (ROC) curve analysis. Patients were categorized into either the culture-positive group (82 cases) or the culture-negative group (69 cases) according to blood culture results.

**Results:**

No significant differences in age and gender were found between the culture-positive and culture-negative groups. The primary bacterial pathogens of bacteremia in the elderly were *Escherichia coli*, *Klebsiella pneumoniae* and *Streptococcus*. Elderly female patients demonstrated a significantly higher culture positivity rate for *E. coli* compared to their male counterparts (*P* = 0.021). The areas under the ROC curve (AUC) for the four parameters were as follows: WBC, 0.851 (95% confidence interval (CI) 0.790 - 0.912); NLR, 0.919 (95% CI 0.875 - 0.963); PLR, 0.609 (95% CI 0.518 - 0.700); and RDW was 0.626 (95% CI 0.563 - 0.717).

**Conclusions:**

*E. coli* was identified as the predominant pathogenic microorganism causing bacteremia in the elderly, with a significantly higher culture positivity rate among female patients. Routine blood parameters (WBC, NLR, PLR, and RDW) demonstrated a predictive potential for diagnosing bacteremia in elderly patients.

## Background

Bacteremia, which can progress to sepsis, is a life-threatening organ dysfunction caused by a disorder in the host’s response to infection, resulting in high morbidity and mortality worldwide (Font et al., 2020; Jirak et al., 2022; Xu et al., 2024). Globally, bacteremia is responsible for approximately 8 million deaths annually, as pathogenic bacteria invade the bloodstream, multiply, and trigger a systemic inflammatory response that often escalates rapidly (Fernando et al., 2018). The most common bacterial causes of bacteremia worldwide include *Escherichia coli*, *Klebsiella pneumoniae*, and *Staphylococcus aureus*, representing both Gram-negative and Gram-positive pathogens (Gatica et al., 2023). Regionally, studies from Asia, particularly China, have highlighted the predominance of Gram-negative bacteria, such as *E. coli* and *K. pneumoniae* as the primary pathogens causing bacteremia in elderly patients. In Chinese investigations, *E. coli* accounted for 47% of bloodstream infections, with *K. pneumoniae* contributing to over 15% (Fu et al., 2024; Li et al., 2024). Similarly, in the United States, *E. coli* has been identified as the leading cause of community-onset bacteremia in seniors, with an estimated 53,476 cases annually among noninstitutionalized elderly individuals (Jackson et al., 2005). These findings highlight the global and regional dominance of these pathogens, particularly in hospitalized or immunocompromised elderly populations.

Bacteremia in elderly patients is particularly concerning due to their reduced immunity and often insidious disease progression, which often results in subtle clinical symptoms, delayed diagnosis, poor prognosis, and higher long-term mortality (Hernández-Quiles et al., 2022). Early detection and intervention are critical in this population to reduce mortality. Currently, the clinical “gold standard” for diagnosing bacteremia is a laboratory blood culture. However, this method has several limitations, including a prolonged processing time (5-7 days), risk of contamination, and potential false-negative results, which can delay treatment and increase mortality (Bonnet et al., 2024).

In recent years, medical practitioners have explored more rapid biomarkers for diagnosing bacteremia, including procalcitonin (PCT), C-reactive protein (CRP), and interleukin-6 (IL-6) (Bonnet et al., 2024). Although these biomarkers can aid in early detection, their moderate diagnostic accuracy, high cost, and lengthy turnaround time limit their widespread application. In contrast, routine blood parameters, which are cost-effective, easy to perform, and readily available in primary healthcare settings, have been proposed as practical tools for predicting bacteremia in high-risk populations (Berhane et al., 2019). Numerous global studies have highlighted the value of routine blood parameters, including white blood cell count (WBC), neutrophil-to-lymphocyte ratio (NLR), platelet-lymphocyte ratio (PLR), and red blood cell distribution width (RDW), in identifying patients at risk of bacteremia and predicting poor outcomes (Agnello et al., 2021). These biomarkers are cost-effective, easily accessible, and hold potential for detecting bacteremia and assessing its severity, especially in resource-limited settings.

Despite these global and regional findings, there is limited understanding of how routine blood parameters correlate with bacteremia in elderly patients, particularly in the Chinese population. In China, few studies have specifically investigated the relationship between routine blood parameters and bacteremia pathogens in elderly patients. Therefore, this study aims to address this gap by investigating the association between routine blood parameters (WBC, NLR, PLR, and RDW) and bacteremia in elderly patients in a Chinese hospital setting. It also examines the distribution of bacterial pathogens in elderly bacteremia patients and evaluates the potential of these routine blood markers as predictive tools for early diagnosis.

## Materials and methods

### Patient characteristics

The clinical and laboratory data of 151 consecutive inpatients aged over 60 years who underwent blood culture testing were retrospectively analyzed between October 2022 and June 2023 at the Department of Clinical Laboratory, Fuding Hospital, Fujian University of Traditional Chinese Medicine. The study focused on elderly patients presenting with suspected bacteremia. The patients were categorized into two groups: the bacteremia group (n = 82), consisting of patients with confirmed positive blood culture results, and the control group (n = 69), consisting of patients with negative blood culture results but similar clinical presentations to ensure a valid comparison.

### Inclusion and exclusion criteria

#### Inclusion criteria

Patients diagnosed with bacteremia based on positive blood culture results, including those who developed sepsis or septic shock during hospitalization, as defined by the Surviving Sepsis Campaign Guidelines (Evans et al., 2021).

#### Exclusion criteria

1. Antibiotic exposure: Patients who received antibiotics more than 24 hours prior to screening were excluded to prevent false-negative blood culture results caused by prior antimicrobial therapy.
2. Pregnant women: Excluded to eliminate the confounding effects of pregnancy-related physiological changes on hematological indices.
3. Immune-compromised patients: Patients receiving radiation therapy, cytotoxic drugs, or organ transplants, as well as those with AIDS or inherited immunodeficiency diseases, were excluded due to altered immune responses that could affect pathogen detection and biomarker reliability.
4. Chronic kidney and liver disease: Patients with chronic kidney disease (baseline serum creatinine ≥2 mg/dL) or chronic liver failure (Child-Pugh grade C) were excluded because these conditions influence inflammatory markers.
5. Long-term treatment cases: Patients with bacterial endocarditis requiring prolonged antibiotic treatment were excluded to focus on acute bacteremia cases.
6. Hematologic and systemic conditions: Patients with conditions affecting hematological indices were excluded, including: Hematologic diseases (e.g., anemia, leukemia); tumors; autoimmune diseases; trauma.

### Ethical approval and compliance

The study protocol was approved by the Medical Ethics Committee of Fuding Hospital, Fujian University of Traditional Chinese Medicine (Approval No.: Fuding Hospital 2022325). All procedures were conducted in accordance with relevant guidelines and regulations. Due to the retrospective nature of the study, the requirement for written informed consent was waived by the ethics committee.

### Laboratory analysis

Blood samples (2.0 mL) were drawn from the median elbow vein of patients prior to initiating treatment, using EDTA as an anticoagulant. Prior to venipuncture, the skin was disinfected thoroughly with 75% alcohol followed by iodine solution to minimize contamination. Complete blood cell counts were analyzed using the SYSMEX XE 2100 fully automated hematology analyzer (Sysmex, Japan) and associated reagents. NLR and PLR were calculated based on neutrophil count, lymphocyte count, and platelet count obtained from routine blood tests. All procedures followed standard operating procedures (SOPs), with in-house quality control conducted to ensure accuracy and reproducibility.

Two sets of bilateral double vials were collected for blood culture, with both aerobic and anaerobic cultures performed simultaneously. Blood samples were obtained via peripheral venous puncture, injected directly into Bactec vials, and incubated at 37°C for up to 7 days in a Bactec incubator (BD Diagnostics, Franklin Lakes, NJ, USA). Positive cultures were immediately Gram-stained and subcultured on solid media for further analysis. Microbial identification was conducted using the VITEK 2 Compact system (bioMérieux, France) for identifying *Enterobacteriaceae* and other Gram-negative bacteria, supplemented by the Vitek mass spectrometry system for precise bacterial species identification. These systems provide high reliability, with pathogen detection accuracy exceeding 95%.

A positive blood culture result from a single vial was considered indicative of bacterial presence. For isolates such as coagulase-negative *Staphylococcus*, *Propionibacterium*, and *Corynebacterium*, the same strain needed to be consistently isolated from multiple samples or repeatedly at the same time; otherwise, it was considered a contaminant from skin colonization.

The study focused on bacterial pathogens isolated from blood cultures. Fungi, *Mycoplasma, Chlamydia, parasites*, and viruses were excluded to maintain the study’s focus on bacterial infections and ensure consistency in statistical analysis.

### Statistical analysis

Data normality was assessed using the Shapiro-Wilk test. Non-normally distributed variables were presented as median and quartiles (P25-P75) and analyzed using nonparametric tests: the Mann-Whitney U test for two-group comparisons and the Kruskal-Wallis H test for multiple groups. Normally distributed data were reported as mean ± standard deviation, and the t-test was employed for mean comparisons between groups. Categorical variables were measured using the chi-square test (χ^2^) or Fisher’s exact tests, as appropriate, and were reported as count (%). Receiver operating characteristic (ROC) curve analysis was conducted for blood routine parameters. The area under the ROC curve (AUC) was calculated along with the standard error and 95% confidence interval (CI). Sensitivity, specificity, accuracy, positive predictive value, and negative predictive value were derived from the ROC curves. Statistical analyses were performed using SPSS 22.0 software (IBM SPSS, Armonk, NY, USA). A P-value of <0.05 was considered statistically significant.

## Results

The Shapiro-Wilk test was used to assess data normality within each group. In all groups, the *P*-value were less than 0.05, indicating non-normal distribution. Consequently, the data were summarized as medians and interquartile ranges. No statistically significant differences in age or gender were observed between the two groups (*P* > 0.05). Detailed results are presented in **Table 1**.

**Table 1:** Clinical baseline parameters of blood culture positive and negative groups [median (P25-P75)].

Groups (cases)	Age (years)	Male (%)	Female (%)
Positive (82)	73.0 (70.0–80.3)	38 (46.3)	44 (53.7)
Negative (69)	72.0 (68.5–75.5)	34 (49.3)	35 (50.7)
Mann-Whitney U/*χ* ^2^ test	-1.793	0.129	
*P*-value	0.073	0.719	

Age is presented as median (25th percentile - 75th percentile). Gender distribution (Male and Female) is presented as number of cases (percentage). Mann-Whitney U test was used for age comparisons, and the Chi-square (χ²) test was used for gender comparisons.

### Analysis of WBC, NLR, PLR, and RDW measurements

The positive group demonstrated significantly higher WBC, NLR, PLR, and RDW values compared to the negative control group, with statistically significant differences (*P* < 0.05). Detailed results are presented in **Table 2**.

**Table 2:** Comparison of blood cell parameters between the two groups [median (P25-P75)].

Parameter	Positive group (n=82)	Negative group (n=69)	Z-value	P-value
WBC (10^9^/L)	10.93 (7.32 – 16.23)	5.96 (4.80–7.18)	-10.407	0.000
Neutrophils (10^9^/L)	8.58 (5.67 – 14.75)	3.69 (2.76–4.54)	-8.407	0.000
Lymphocytes 10^9^/L)	1.00 (0.59 - 1.49)	1.70 (1.38–2.18)	-6.307	0.000
RDW (%)	13.70 (13.00-15.00)	12.90 (12.30- 14.00)	-2.676	0.007
NLR	10.52 (5.50–18.44)	2.01 (1.36–3.07)	-8.853	0.000
PLR	188.61 (97.64–250.00)	135.86 (97.66–187.50)	-2.312	0.021

Non-parametric comparisons between the two groups was performed using the Mann-Whitney U test. WBC, White blood cells; NLR, Neutrophil-to-lymphocyte ratio; PLR, Platelet-to-lymphocyte ratio; RDW, Red cell distribution width.

### Distribution and composition of bacterial pathogens

A total of 82 bacterial strains were isolated from the blood samples of 82 hospitalized elderly patients. The prevalence of Gram-negative bacteria was significantly higher among elderly female patients with bacteremia (61 cases, 74.4%) compared to their male counterparts (21 cases, 25.6%) (*P* = 0.021) (**Table 3**). Fungal infections were excluded from the analysis.

**Table 3:** Distribution of pathogenic bacteria between male and female groups (n, %).

Pathogen (Strains)	Male (n = 37)	Female (n = 45)	χ²	*P*- value
Gram-stain (n = 82)				
Positive (n = 21)	14 (37.8)	7 (15.6)	5.292	0.021a
Negative (n = 61)	23 (62.2)	38 (84.4)		
*Escherichia coli* (n = 42)				
Positive	13 (35.1)	29 (64.4)	6.981	0.008a
Negative	24 (64.9)	16 (35.6)		
*Klebsiella pneumoniae* (n = 15)				
Positive	9 (24.3)	6 (13.3)	1.641	0.200a
Negative	28 (75.7)	39 (86.7)		
*Streptococcus* (n = 10)				
Positive	7 (18.9)	3 (6.7)	–	0.089b
Negative	30 (81.1)	42 (93.3)		
*Staphylococcus* (n = 9)				
Positive	5(13.5)	4 (8.9)	–	0.725b
Negative	32 (86.5)	41 (91.1)		
Other bacteria (n = 6)				
Positive	3 (8.1)	3 (6.7)	–	1.000b
Negative	34 (91.9)	42 (93.3)		

aChi-square test. bFisher’s exact test. Other bacteria include *Enterococcus faecium* (2 strains), *Bacteroides fragilis* (2 strains), *Proteus mirabilis* (1 strain), *Moraxella osloensis* (1 strain).

**Figure 1:**
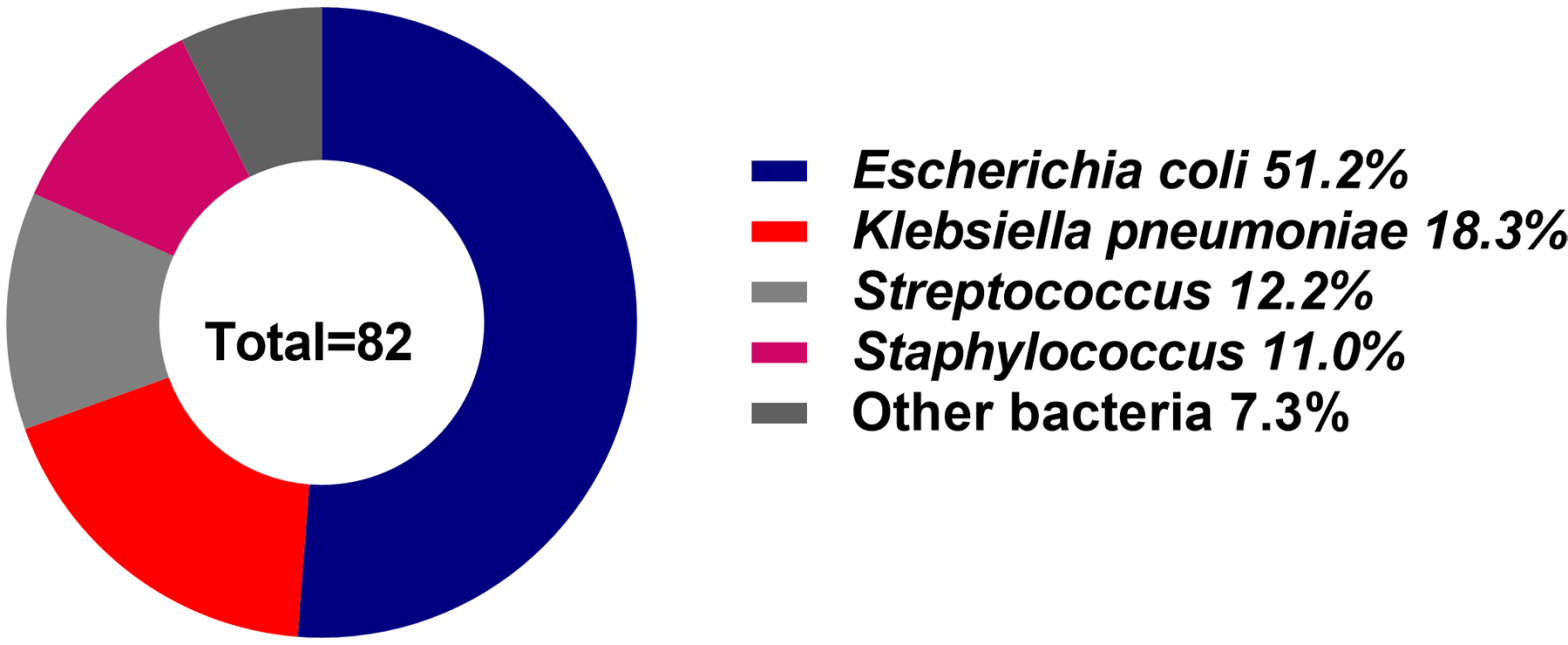
Distribution of bacterial pathogens in elderly patients with bacteremia. *Escherichia coli* was the most prevalent pathogen (51.2%), followed by *Klebsiella pneumoniae* (18.3%), *Streptococcus* (12.2%), and others (18.3%).

The distribution of bacterial pathogens included 42 strains of *E. coli*, 15 strains of *K. pneumoniae*, 10 strains of *Streptococcus*, and 9 strains of *Staphylococcus aureus*. Additionally, six strains of other bacteria accounted for 7.3% of the isolates, comprising 2 strains of *Enterococcus faecium*, 2 strains of *Bacteroides fragilis*, 1 strain of *Proteus mirabilis*, and 1 strain of *Moraxella osloensis.* Detailed data are presented in **Table 4** and **Figure 1**.

**Table 4:** Kruskal-Wallis H test of routine blood parameters across different pathogens.

Pathogen (Strains)	Number Rank Average			
	WBC	NLR	PLR	RDW
*Escherichia coli* (42)	43.79	38.90	41.48	40.90
*Klebsiella pneumoniae* (15)	43.90	48.80	39.60	36.23
*Streptococcus* (10)	32.30	37.50	43.00	35.70
*Staphylococcus* (9)	33.06	42.78	44.11	57.61
Other bacteria (6)	47.50	46.17	40.00	44.33
H-value	3.544	2.447	0.267	5.618
p-value	0.471	0.654	0.992	0.230

The non-parametric test Kruskal-Wallis H test was used to compare the five groups of routine blood parameters across different pathogens. No statistically significant differences were observed for any parameter (all *P* > 0.05). As a result, the Bonferroni method was not applied for *post-hoc* pairwise comparisons. WBC, White blood cell count; NLR, Neutrophil-to-lymphocyte ratio; PLR, Platelet-to-lymphocyte ratio; RDW, Red cell distribution width. Other bacteria: *Enterococcus faecium* (2 strains), *Bacteroides fragilis* (2 strains), *Proteus mirabilis* (1 strain), *Moraxella osloensis* (1 strain).

**Tables 1**–**4** present detailed statistical analyses comparing the study groups and summarizing key outcomes. These tables highlight the characteristics of the groups, statistical significance, counts of various cell types, and the effectiveness of routine blood parameters. Specially, **Table 4** demonstrates the results of the Kruskal-Wallis H test, which examines differences among various pathogens and their associations with routine blood parameters. Additionally, **Table 5** and **Figure 2** illustrate the ROC curve analysis, providing optimal cutoff values, sensitivities, specificities, accuracies, and predictive values for different blood cell parameters.

**Table 5:** Area under the receiver operating characteristic (ROC) curve and related parameters.

Item	WBC	NLR	PLR	RDW
Optimal Cutoff	8.0	4.0	205.9	13.0
Youden index (%)	61.3	69.4	26.7	30.2
Sensitivity (%)	74.4	85.4	42.7	78.0
Specificity (%)	87.0	84.1	84.1	52.2
Accuracy (%)	80.1	84.8	61.6	66.2
PPV (%)	87.1	86.4	76.1	66.0
NPV (%)	74.1	82.9	55.2	66.7
AUC (95%CI)	0.851 (0.790-0.912)	0.919 (0.875-0.963)	0.609 (0.518-0.700)	0.626 (0.536-0.717)
Standard Error	0.031	0.022	0.046	0.046
*P*- value	0.000	0.000	0.021	0.008

AUC, Area under the receiver operating characteristic (ROC) curve; 95%CI, 95% confidence interval; WBC, White blood cells; NLR, Neutrophil-to-lymphocyte ratio; PLR, Platelet-to-lymphocyte ratio; RDW, Red cell distribution width; PPV, Positive predictive value; NPV, Negative predictive value.

**Figure 2:**
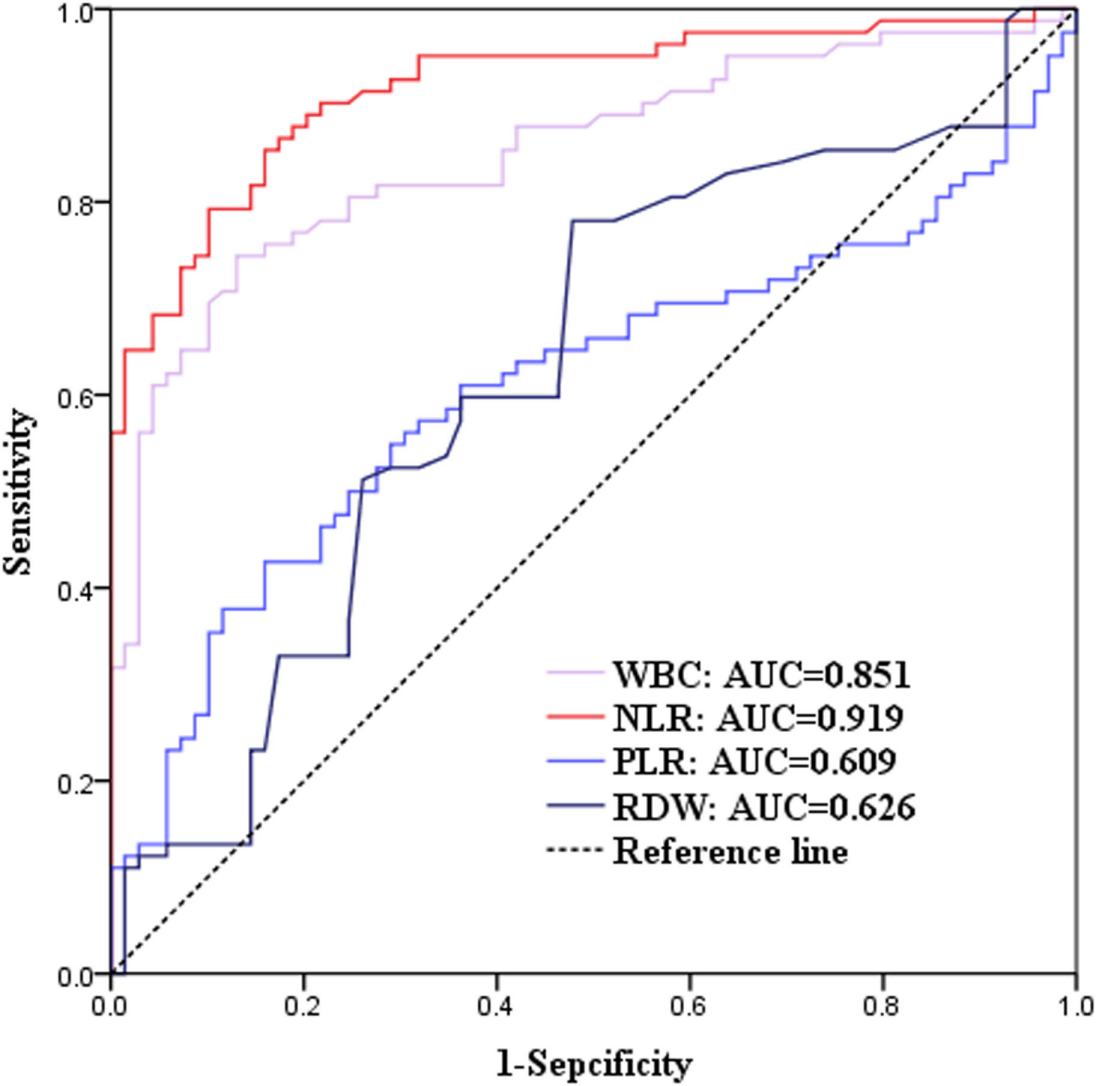
Receiver operating characteristic (ROC) curves for blood parameters. The area under the curve (AUC) values were: White blood cell (WBC): 0.851; Neutrophil-to-lymphocyte ratio (NLR): 0.919; Platelet-to-lymphocyte ratio (PLR): 0.609; Red blood cell distribution width (RDW): 0.626. Optimal cutoff values, sensitivity, and specificity derived from the ROC analysis highlight the diagnostic performance of each parameter.

## Discussion

This retrospective study of 82 elderly patients with bacteremia and 69 controls identified *E. coli* (51.2%), *K. pneumoniae* (18.3%), and *Streptococcus* (12.2%) as the most prevalent bacteria pathogens (**Figure 1**). These findings align with previous studies, including those by Guarno et al. (Guarino et al., 2023) and Daniela Dambroso et al (Dambroso-Altafini et al., 2022), which highlighted reported Gram-negative bacteria, particularly *E. coli* and *K. pneumoniae*, as leading causes of bloodstream infections. Similarly, a Japanese study (Matono et al., 2022) reported comparable trends, identifying *E. coli* (28/58, 48%) and *K. pneumoniae* (6/*58*,10%) as the predominant pathogens.

Our findings reflect global trends where *E. coli* predominates in elderly individuals due to its frequent colonization of the gastrointestinal tract, a critical infection focus for bloodstream infections. Disruptions in intestinal barrier integrity facilitate the translocation of gut-resident *E. coli* into the bloodstream, contributing to systemic infections (Tao et al., 2024; Wu et al., 2024). Identifying key infection foci, such as the gastrointestinal and urinary tracts, is vital for improving clinical management and preventing sepsis progression. In our study, *E. coli* demonstrated a significantly higher positivity rate in elderly women compared to men (64.4% vs. 35.1%, *P* = 0.008) (**Table 3**). This gender disparity is likely attributed to anatomical factors, including a shorter urethra in females, which increases their susceptibility to urinary tract infections (UTIs)—a common source of *E. coli* bacteremia in this population (Poolman and Wacker, 2016; Plasencia and Ashraf, 2024; Zhan et al., 2024).

Beyond UTIs, *E. coli* can invade human tissues, causing severe infections and high mortality (Fay et al., 2020). It remains the leading cause of bacteremia globally and a major contributor to sepsis-related hospitalizations and deaths among elderly patients (Bonten et al., 2021). Mortality rates in this population range from 15% to 30%, highlighting the critical need for early detection and timely intervention (Tocut et al., 2022).

The analysis of blood cell parameters showed significant increases in leukocyte count, NLR, PLR, and RDW among bacteremia patients. Among these, NLR demonstrated the highest predictive value, with a sensitivity of 85.4% and specificity of 84.1% (**Figure 2**). Elevated WBC, PLR, and RDW were also significantly associated with bacteremia. These findings underscore the potential of routine blood parameters as rapid, cost-effective, and accessible diagnostic tools, especially in resource-limited settings where immediate blood culture results are unavailable.

While WBC is traditionally used as an indicator of bacterial infection, its diagnostic accuracy may be influenced by conditions such as hematological diseases, non-infectious inflammatory conditions, surgery, and trauma (Wu et al., 2023). RDW, a marker for red blood cell size variability, is primarily used to diagnose anemia (Liu et al., 2023). However, in conditions like sepsis, oxidative stress and inflammation disrupt erythrocyte maturation, resulting in elevated RDW levels (Jain et al., 2022). A study by Dogan P et al. reported that an RDW cutoff of >19.50% had a sensitivity of 87% and specificity of 81% for predicting late-onset Gram-negative sepsis. In contrast, our study identified an RDW cutoff of >13.0%, achieving a sensitivity of 78.0% and specificity of 52.2%, likely reflecting differences in patient populations.

Our study highlights the value of NLR as a reliable indicator of systemic inflammatory response and a strong predictor of bacteremia (Agnello et al., 2021). Routine blood parameters, including NLR, PLR, and RDW, represent valuable diagnostic tools due to their accessibility, cost-effectiveness, and rapid turnaround time. Clinically, these parameters can aid in the early identification of bacteremia in elderly patients, particularly in resource-limited settings where immediate blood culture results may not be available. Furthermore, While this study focus on the diagnostic performance of blood parameters and pathogen distribution, further research should investigate the relationship between clinical outcomes, blood parameters, and drug susceptibility patterns of the detected pathogens. Additionally, identifying infection foci, particularly in sepsis cases, may further improve clinical management and outcome prediction. Future studies addressing these aspects will provide a more comprehensive understanding of bacteremia and its clinical implications.

## Conclusion

Our cross-sectional study identified *E. coli* as the predominant pathogen in elderly patients with bacteremia, with a higher positivity rate in females. This finding underscores the importance of investigating urinary and gastrointestinal infection foci in this demographic. Routine blood parameters, particularly WBC, NLR, PLR, and RDW, showed significant predictive value for diagnosing bacteremia. These accessible and cost-effective markers can facilitate early detection and timely treatment, particularly in resource-limited settings, improving outcomes for at-risk elderly patients. Future research should examine the relationship between blood parameters, clinical outcomes, and pathogen drug susceptibility to enhance diagnostic accuracy and optimize treatment strategies.

## Limitations

This study focused exclusively on bacterial pathogens, excluding others such as fungi, *Mycoplasma*, *Chlamydia*, parasites, and viruses. Future research should adopt a multi-center design with larger sample sizes to offer a more comprehensive understanding of geriatric bacteremia. Additionally, further investigations are needed to explore microorganism distribution and enhance the predictive utility of routine blood parameters.

## Data Availability

The raw data supporting the conclusions of this article will be made available by the authors, without undue reservation.
